# Flavonoid Naringenin: A Potential Immunomodulator for *Chlamydia trachomatis* Inflammation

**DOI:** 10.1155/2013/102457

**Published:** 2013-05-23

**Authors:** Abebayehu N. Yilma, Shree R. Singh, Lisa Morici, Vida A. Dennis

**Affiliations:** ^1^Department of Biological Sciences, Center for NanoBiotechnology and Life Sciences Research (CNBR), Alabama State University, 1627 Hall Street, Montgomery, AL 36104, USA; ^2^Department of Microbiology and Immunology, Tulane University School of Medicine, 1430 Tulane Avenue, SL-38, New Orleans, LA 70112, USA

## Abstract

*Chlamydia trachomatis*, the agent of bacterial sexually transmitted infections, can manifest itself as either acute cervicitis, pelvic inflammatory disease, or a chronic asymptomatic infection. Inflammation induced by *C. trachomatis* contributes greatly to the pathogenesis of disease. Here we evaluated the anti-inflammatory capacity of naringenin, a polyphenolic compound, to modulate inflammatory mediators produced by mouse J774 macrophages infected with live *C. trachomatis*. Infected macrophages produced a broad spectrum of inflammatory cytokines (GM-CSF, TNF, IL-1**β**, IL-1**α**, IL-6, IL-12p70, and IL-10) and chemokines (CCL4, CCL5, CXCL1, CXCL5, and CXCL10) which were downregulated by naringenin in a dose-dependent manner. Enhanced protein and mRNA gene transcript expressions of TLR2 and TLR4 in addition to the CD86 costimulatory molecule on infected macrophages were modulated by naringenin. Pathway-specific inhibition studies disclosed that p38 mitogen-activated-protein kinase (MAPK) is involved in the production of inflammatory mediators by infected macrophages. Notably, naringenin inhibited the ability of *C. trachomatis* to phosphorylate p38 in macrophages, suggesting a potential mechanism of its attenuation of concomitantly produced inflammatory mediators. Our data demonstrates that naringenin is an immunomodulator of inflammation triggered by *C. trachomatis*, which possibly may be mediated upstream by modulation of TLR2, TLR4, and CD86 receptors on infected macrophages and downstream via the p38 MAPK pathway.

## 1. Introduction

Sexually transmitted *Chlamydia trachomatis* infection is of widespread public health concern because of its prevalence and potentially devastating reproductive consequences, including pelvic inflammatory disease (PID), infertility, and ectopic pregnancy [1–3]. The negatively charge elementary bodies (EB), infectious particles of *C. trachomatis*, invade the mucosal surface of the female genital tract and persist in them for a long time [2]. Abundant *in vitro* data suggests that the inflammatory response to Chlamydiae is initiated and sustained by actively infected host cells including epithelial cells and resident macrophages [4].

*C. trachomatis* has the ability to infect both epithelial cells and resident macrophages. These infected host cells act as first responders to initiate and propagate immune responses, which later participate in initiation of adaptive immune responses. Activation of adaptive immune responses consequently leads to accumulation of effector T and B cells at the site of *Chlamydia* infection and plays critical roles in controlling the infection [5, 6]. However, *C. trachomatis* uses various strategies to escape the host immune response and persist for a prolonged period of time, subsequently leading to the many disease manifestations associated with the infection. This is a common scenario for most intracellular organisms such as *Mycobacteria*, where cells produce excessive inflammatory mediators to contribute to disease manifestation by damaging neighboring cells [7]. For example, results from studies using the murine model of *C. trachomatis* revealed that tubal dilation frequently occurred as an end result for a primary infection, suggesting that the inflammatory process resulting from a single *C. trachomatis* infection is sufficient to result in long-term tissue damage [8].

Like other infectious microorganisms, inflammatory mediators have been documented to be hallmarks of *C. trachomatis* infection and its pathogenesis [4–6]. Because of the inherent difficulties in acquiring human tissue samples for study, researchers have taken advantage of multiple animal models of *Chlamydia* infection to examine the nature and timing of the inflammatory response. We have shown by *in vitro *experiments that primary *Chlamydia* infection of human epithelial cells and mouse macrophages occurs within 2 days of infection and is characterized by significant production of IL-6, TNF, and IL-8 [9]. It is well documented that inflammatory cytokines and chemokines play critical role for the recruitment and chemoattractant of neutrophils and other leukocytes. Neutrophils have the capability to destroy accessible EBs, and when recruited in high numbers, they release matrix metalloprotease (MMPs) molecules and neutrophil elastase, which have been shown to contribute to tissue damage [10, 11].

To control inflammation triggered by infectious organisms, alternative strategies that could balance the levels of inflammatory mediators released during infection are of intense interest. Recently active compounds with the capacity to modulate host inflammatory responses have received considerable attention as they may be potential new therapeutic agents for the treatment of inflammatory diseases [12–15]. Naringenin is a naturally occurring polyphenolic compound containing two benzene rings linked together with a heterocyclic pyrone ring [16]. Naringenin is a normal constituent of the human diet in grapefruit and tomatoes and is known to exhibit a variety of biological activities, such as enzyme inhibitors, antioxidants, anticancer, and as an anti-inflammatory agent [17–21].

Since its discovery, naringenin's wide ranges of pharmacological properties have attracted the attentions of many researchers because of its anti-inflammatory properties. Its anti-inflammatory property is actively studied in macrophages and *ex vivo* human whole-blood models [22–24]. In this study, we investigated the anti-inflammatory capacity of naringenin to regulate cytokines and chemokines produced by mouse J774 macrophages infected with live *C. trachomatis* (MoPn Nigg II). We used multiplex ELISA to determine a broad range of inflammatory cytokines and chemokines produced during the interaction of *C. trachomatis* and macrophages. We then assessed the ability of naringenin to regulate the production level of these mediators. Next, we determined the potential mechanism(s) by which naringenin may modulate inflammatory mediators by investigating its effect on TLR2, TLR4, and CD86 receptors, as well as the p38 MAPK pathway. The findings from our study are discussed here in the context of naringenin as a potential new immunomodulator of *C. trachomatis* induced inflammation.

## 2. Materials and Methods

### 2.1. Cell Culture and Infectivity

Mouse J774 macrophages were obtained from the American Type Culture Collection (ATCC, Manassas, VA, USA) and cultured as already described [9]. *C. trachomatis* MoPn Nigg II was purchased from ATCC (ATCC VR-123) and propagated as previously described [9]. To establish infection, macrophages (10^6^ cells/well) were seeded in 24-well plates for 24 h after which they were infected with live *C. trachomatis* infectious particles (10^5^) in 500 *μ*L of growth media/well. The cells were then incubated at 37°C under 5% CO_2_ and culture supernatants were collected at 48 h after infection. The optimum bacterium dose and duration of infection were determined as reported [9]. As a positive control, macrophages (10^6^ cells/well) were stimulated with *E. coli* LPS (1 *μ*g/mL) and culture supernatants were collected at 48 h after stimulation. Collected supernatants were centrifuged at 450 ×g for 10 min at 4°C and stored at −80°C until used.

### 2.2. Preparation of Naringenin

The stock solution of naringenin (Sigma, St. Louis, MO, USA) was prepared by dissolving 40 mg of naringenin in 1 mL dimethyl sulfoxide (DMSO). After 2-day infection of macrophages with *C. trachomatis*, the media were replaced with fresh media containing various concentrations (0.01, 0.1, 1, and 10 *μ*g/mL) of naringenin. Cell-free supernatants were collected after an additional 48 h incubation following centrifugation at 450 ×g for 10 min at 4°C and stored at 80°C until used.

### 2.3. Inflammatory Cytokines and Chemokines

Milliplex mouse 32-plex cytokine and chemokines detection reagent (catalogue number MPXMCYTO-70 K-PMX32) was purchased from Millipore (EMD Millipore Corporation, Billerica, MA, USA) and the assay was performed as described [25].

### 2.4. Cytotoxicity Studies

Cytotoxicity of naringenin to mouse J774 macrophages was measured using the 3-(4, 5-dimethylthiazol-2-yl)-2,5-diphenyl tetrazolium bromide (MTT) dye reduction assay and the CellTiter 96 Cell Proliferation Assay kit (Promega, Madison, WI, USA). Cells were seeded in a 96-well plate at a density of 10^5^ cells/well in 50 *μ*L media and incubated overnight at 37°C under 5% CO_2_. Naringenin was added to cells in concentrations ranging from 0.1 to 100 *μ*g/mL and after 48 h supernatants were removed, cells were washed twice with sterile PBS, followed by addition of 15 *μ*L of MTT dye solution to each well, and cells were further incubated for 3 h at 37°C under 5% CO_2_. To stop the reaction, 100 *μ*L of solubilization solution/stop mixture was added to each well and plates incubated for 30 min at room temperature (RT). Absorbance at 570 nm was measured using a Tecan Sunrise plate reader (TECAN US Inc., Durham, NC, USA). The percentage of cell viability was obtained using the optical density readings of naringenin treated cells compared to those of normal cells (control), where % viability = [*A*]_test_/[*A*]_control_ × 100, where [*A*]_test_ is the absorbance of the test sample and [*A*]_control_ is the absorbance of control sample.

### 2.5. Flow Cytometry

Mouse J774 macrophages (10^6^ cells/mL) were left uninfected or infected with *C. trachomatis* and after 48 h infection the media were removed and replenished with fresh media containing 1 *μ*g/mL of naringenin. Following incubation for an additional 48 h, cells were scraped from wells, washed, and then blocked with Fc blocking antibody (BD Bioscience) in FACS (fluorescence-activated cell sorting) buffer (PBS Containing 0.1% NaN_3_ and 1% fetal bovine serum) for 15 min at 4°C. Cells were next washed two times followed by staining with fluorochrome-conjugated antibodies (50 *μ*L in FACS buffer) against mouse TLR2 (PE), TLR4 (FITC), CD80 (PE-Cy7), and CD86 (APC) (eBiosciences). The optimum concentrations of all fluorochromes were predetermined in our laboratory. Cells were incubated with fluorochrome antibodies for 30 min at 4°C, washed 2 times, and then fixed using 2% paraformaldehyde solution. Data were acquired on a BD FACSCanto II flow cytometer (BD Bioscience) with at least 10^5^ events for each sample. TLR2, TLR4, CD80, and CD86 positive cells and their mean fluorescence intensity (MFI) were analyzed using FlowJo software (TreeStar Inc., Ashland, OR, USA).

### 2.6. RNA Extraction and Quantitative Real-Time PCR (qRT-PCR)

Mouse J774 macrophages (3 × 10^6^ cells/well) were infected with live *C. trachomatis* (3 × 10^5^ IFU/well) in 6-well plates for 48 h followed by replacement of fresh media containing 1 *μ*g/mL of naringenin. RNA was extracted from the cell pellets using Qiagen RNeasy Kit (Qiagen Inc., Valencia, CA, USA), which included a DNase-I digestion step. qRT-PCR was employed to quantify mRNA gene transcripts of CD86 and TLR2 using TaqMan RNA-to-C_T_ 1-step kit in combination with TaqMan gene expression assays (Applied Biosystems by Life Technologies, Foster City, CA, USA) as reported [25]. Amplification of gene transcripts was performed according to the manufacturer's protocol using ABI ViiA 7 real-time PCR (Applied Biosystem by Life Technologies) and standard amplification conditions. The relative changes in gene expression were calculated using the following equation: 2^−ΔΔCT^ where all values were normalized with respect to the “housekeeping” gene GAPDH mRNA levels. Amplification using 50 ng RNA was performed in a total volume of 20 *μ*L. Each real-time PCR assay was performed in triplicates and the results are expressed as the mean ± SD.

### 2.7. Inhibition of p38 MAP Kinase Pathway

To determine if the p38 MAPK pathway is employed by *C. trachomatis* to trigger production of cytokines and chemokines by mouse J774 macrophages, we next blocked p38 MAPK signaling with its specific inhibitor, SB203350 (EMD Millipore Corporation, Billerica, MA, USA). Mouse J774 macrophages (10^6^ cells/well) were preincubated with 20 *μ*M of SB203350 for 24 h, infected with *C. trachomatis* (10^5^ IFU/well), and incubated for an additional 72 h. Cell-free supernatants were collected by centrifugation and the production levels of randomly selected cytokines (IL-6, TNF, IL-12p70, and IL-1*β*) and chemokines (CCL5 and CXCL10) were determined using single ELISAs as described previously [9]. The 20 *μ*M concentration and 24 h inhibition time point used for SB203350 were optimal conditions predetermined in our laboratory.

### 2.8. Phosphorylation of p38 MAPK by *C. trachomatis*

Mouse J774 macrophages (3 × 10^6^ cells/well) were seeded in 6-well plates and infected with live *C. trachomatis* (3 × 10^5^ IFU/well) for 15, 30, and 60 min. Cells were lysed at different time points using 1x RIPA buffer (Sigma) supplemented with phosphatase inhibitors (Sigma). Immediately cells were transferred to microcentrifuge tubes, sonicated for 15 sec to shear DNA and reduce sample viscosity followed by centrifugation at 450 g for 10 min at 4°C. The concentrations of proteins were determined by the bicinchoninic acid assay (BCA) (Thermo Scientific, Rockford, IL, USA). Proteins were separated by SDS-PAGE, transferred to nitrocellulose membranes, and blocked with blocking buffer (tris-buffered saline (TBS)) containing 0.1% Tween-20 and 5% w/v nonfat milk. After blocking for 1 h, the membrane was washed 3 times for 5 min each with wash buffer (TBS, 0.1% Tween-20) and incubated overnight with gentle agitation at 4°C with phospho-p38 or total p38 primary antibodies (Cell Signaling Technology Inc., Beverly, MA, USA) each at a dilution of 1 : 1000 (diluted in primary antibody dilution buffer (1x TBS, 0.1% Tween-20, 5% bovine serum album (BSA), and dH_2_O). Following overnight incubation, the membrane was washed 3 times and incubated with HRP-conjugated secondary antibody (Cell Signaling) at 1 : 2000 (diluted in blocking buffer) with gentile agitation for 1 h at RT. After 3 washes, protein bands were visualized using LumiGLO substrate (Cell Signaling) on scientific imaging film (Kodak Inc., Rochester, NY, USA). The sizes of total p38 and phospho-p38 were determined from the biotinylated protein ladder. The optimum concentrations for antibodies were used according to the manufactures suggestion. Biotinylated secondary antibody (1 : 1000 diluted in blocking buffer) was used to detect the protein markers. For some experiments, macrophages were infected with *C. trachomatis* in the presence and absence of naringenin at 1 *μ*g/mL to determine if naringenin may exert its anti-inflammatory activity by blocking the p38 MAPK pathway. Protein lysates were collected and used in western blotting to detect the phosphorylation of p38 MAPK as described in the preceding paragraph.

### 2.9. Statistics Analysis

The two-tailed unpaired Student's *t*-test was used to compare the data. *P* < 0.05 was considered significant.

## 3. Results

### 3.1. The Effect of Naringenin on the Levels of Inflammatory Cytokines and Chemokines Produced by *C. trachomatis* Infected Macrophages

**Figure 1:**
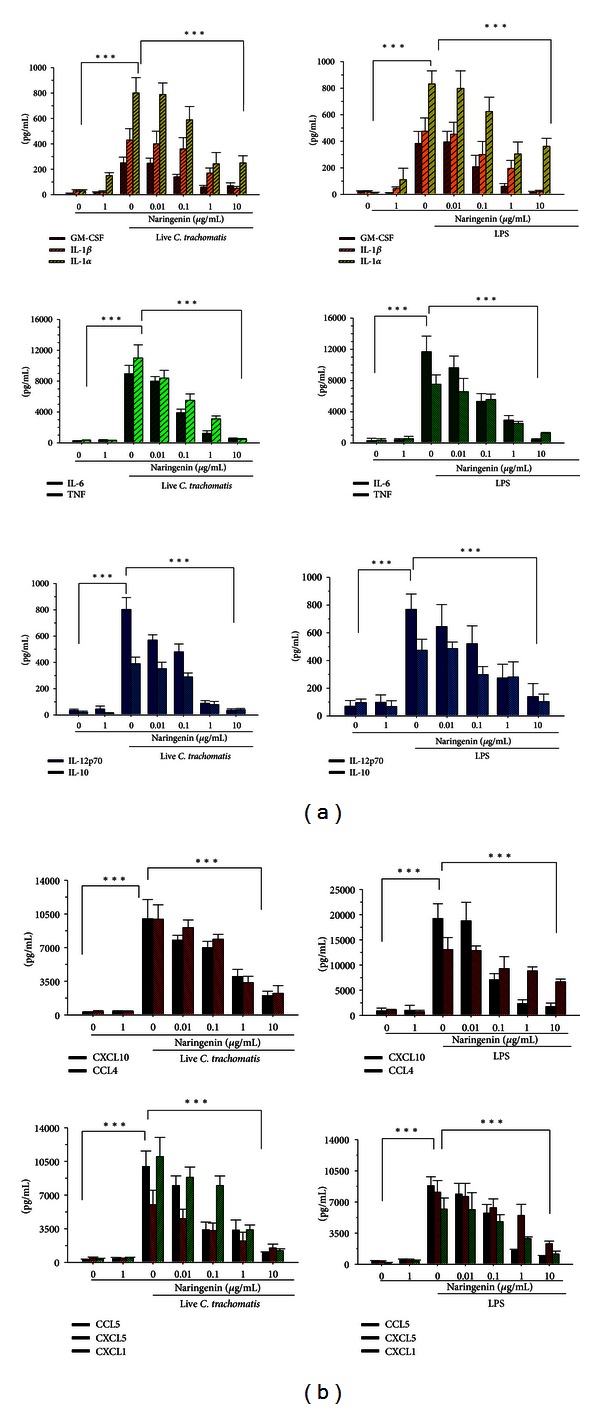
Naringenin downregulates inflammatory mediators in *C. trachomatis* infected mouse J774 macrophages. Macrophages (10^6^ cells/mL) were seeded in 24-well plates and were either infected with live *C. trachomatis* (10^5^ IFU/well) or LPS at 1 *μ*g/mL. After 2-day infection, naringenin at 0.01 to 10 *μ*g/mL was added to cell cultures and the production levels of cytokines (a) and chemokines (b) were quantified in supernatants collected 2 days later employing multiplex ELISA. ***indicates significant difference (*P* < 0.001) between *C. trachomatis* treated cells and those treated with various concentrations of naringenin using the two-tailed unpaired Student's *t*-test. Each bar represents the average of samples run in duplicates and the data are representative of three separate experiments.

Like other infection agents,* C. trachomatis* induces the secretion of various inflammatory mediators upon its infection of macrophages. In the present study, we employed multiplex ELISA to identify and quantify cytokines and chemokines in supernatants from macrophages infected with live *C. trachomatis*. Infected macrophages produced significant (*P* < 0.001) levels of cytokines (IL-6, TNF, IL-10, IL-12p70, IL-1*α*, IL-1*β*, and GM-CSF) and chemokines (CCL4, CXCL10, CXCL5, CCL5, and CXCL1) (Figures 1(a) and 1(b)). However, the production levels of these mediators were reduced in a dose-dependent manner in the presence of added naringenin (Figures 1(a) and 1(b)). Supernatants of *C. trachomatis* infected macrophages that contained 10 *μ*g/mL of added naringenin showed a significant reduction in the levels of cytokines and chemokines (*P* < 0.001) (Figures 1(a) and 1(b)). The inhibitory activity of naringenin was significantly (*P* < 0.01) observed with as little as 1 *μ*g/mL (Figure 1(a)), suggesting the potency of naringenin even at low concentrations. Naringenin similarly reduced the production levels of cytokines and chemokines in a dose-dependent manner (*P* < 0.001) when LPS was used as the stimulant, especially at 10 *μ*g/mL (Figures 1(a) and 1(b)). Overall, our results indicate that naringenin has an anti-inflammatory effect against *C. trachomatis* induced inflammatory mediators by macrophages.

### 3.2. The Anti-Inflammatory Effect of Naringenin Is Not due to Cell Death

**Figure 2:**
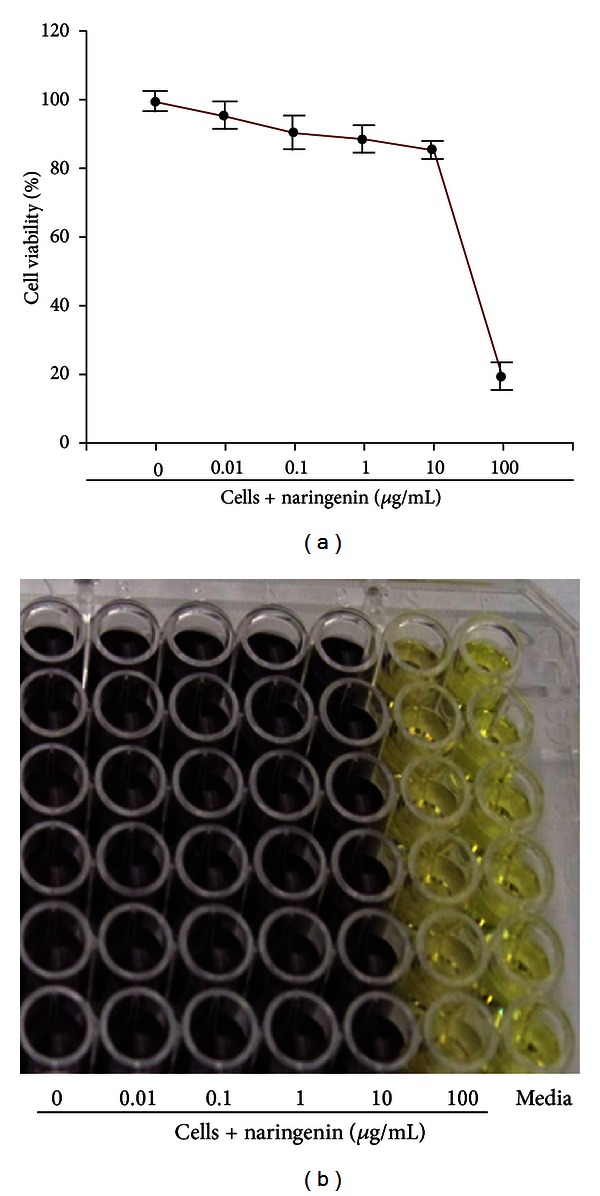
Naringenin toxicity to mouse J774 macrophages is concentration dependent. Macrophages were seeded in a 96-well plate at a density of 10^5^ cells/well/50 *μ*L in the presence or absence of naringenin in concentrations ranging from 0.1 to 100 *μ*g/mL. The CellTiter 96 Cell Proliferation Assay kit was used to determine cell viability. Absorbance was read at 570 nm and the percentage of cell viability was calculated by using the optical density readings compared to normal cells (a). A representative plate before the absorbance readings where dark purple and yellow wells are depictions of live and dead cells, respectively (b). The data are representative of three separate experiments.

To ensure that the inhibitory effect of naringenin is not attributed to cell death, cytotoxicity studies were performed employing the MTT assay and J774 macrophages exposed to various concentrations of naringenin (0.01 to 100 *μ*g/mL). With the exception of the 100 *μ*g/mL naringenin concentration, all other tested concentrations exhibited between 85% and 100% cell viability, suggesting that naringenin is effectively nontoxic to macrophages at these concentrations (Figure 2(a)). Figure 2(b) depicts a representative 96-well plate with cell death occurring in the presence of 100 *μ*g/mL of naringenin (yellow color) versus viable cells at other naringenin concentrations (dark purple color). Overall, these results demonstrate that naringenin's anti-inflammatory effect on inflammatory mediators produced by *C. trachomatis* infected macrophages is not attributed to cell death but rather to alternative mechanisms.

### 3.3. Naringenin Downregulates the Expression Levels of CD86, TLR2, and TLR4 on J774 Macrophages

**Figure 3:**
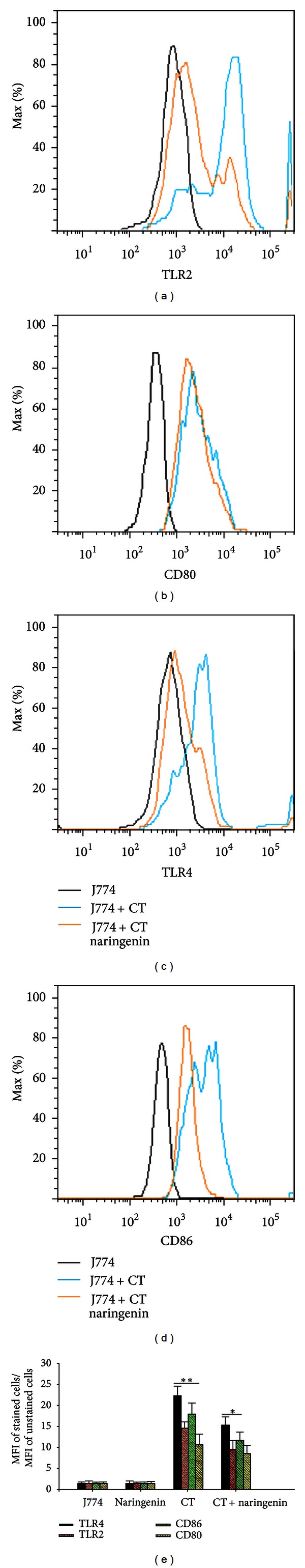
Naringenin downregulates the expression levels of CD80, TLR2, and TLR4 in *C. trachomatis* infected mouse J774 macrophages. Macrophages (10^6^ cells/mL) were seeded in 24-well plates and infected with *C. trachomatis* (10^5^ IFU/well) or left uninfected. After 2-day infection, 1 *μ*g/mL naringenin was added to cell cultures and 2 days later samples were analyzed by flow cytometry as described in Section 2. Shown are the expression shifts of TLR2 (a), CD80 (b), TLR4 (c), and CD86 (d) and their mean fluorescence intensity (MFI) (e) before and after infection of macrophages with *C. trachomatis* (CT) in the presence and absence of naringenin. *P < 0.05 is considered significant as compared to untreated cells (J774) and to cells treated with CT or CT + naringenin. The data are representative of two separate experiments.

Receptors on host cell surfaces such as TLRs recognize extracellular stimuli for subsequent intracellular signaling processes. Multiple studies have shown that TLR2 and TLR4 play pivotal roles in the recognition of *C. trachomatis* [26–29]. To begin to understand the mechanism(s) by which naringenin modulates inflammatory mediators, we first focused on whether or not naringenin will affect the putative TLR2 and TLR4 receptors expressed on *C. trachomatis* infected mouse J774 macrophages. As compared to unstimulated cells, *C. trachomatis* infected cells expressed more TLR2 and TLR4 receptors, which were markedly downregulated in the presence of added naringenin, especially for TLR2 (Figures 3(a) and 3(c)). In addition, the MFI for TLR2 and TLR4 on* C. trachomatis* infected cells was significantly increased (*P* < 0.05) as shown by ratios of 22 and 16, respectively, in comparison to those of J774 and naringenin only uninfected cells (Figure 3(e)). When naringenin was added to *C. trachomatis* infected macrophages, the MFI of TLR2 and TLR4 reduced significantly (*P* < 0.05) as compared with that of *C. trachomatis* infected macrophages (Figure 3(e)), suggesting the ability of naringenin to down-regulate the expression of these receptors. Our result provides evidence that naringenin diminishes the recognition of *C. trachomatis* by its putative TLR2 and TLR4 receptors to possibly exert its anti-inflammatory downstream effects during reinfection of cells by *C. trachomatis*.

Activated T cells produce additional inflammatory cytokines and chemokines to direct immune responses. For T cells to be fully activated, antigen presenting cells must express costimulatory molecules such as CD80 and CD86 [30]. Therefore, down-regulating the expression of either CD80 or CD86 or both may negatively impact the activation of T cells. Here we tested if naringenin may impact T-cell activation by down-regulating CD80 and CD86 expression levels on* C. trachomatis* infected macrophages. Our flow cytometric results show that naringenin at 1 *μ*g/mL downregulates the expression of CD86 induced by *C. trachomatis* infected macrophages but not that of CD80 as compared to macrophages exposed only to *C. trachomatis* (Figures 3(b) and 3(d)). Moreover, naringenin significantly reduced (*P* < 0.05) the MFI of CD86 on *C. trachomatis* infected cells from 18 to 9 (Figure 3(e)). On the other hand, naringenin did not reduce the MFI of CD80 on infected cells (Figure 3(e)), indicating its selective modulation of costimulatory molecules on *C. trachomatis* infected cells. This finding further suggests that naringenin anti-inflammatory effect is not only limited to innate immune responses but also to adaptive immune responses since the expression of either CD80 or CD86 or both plays critical roles for activation of T cells during adaptive immune responses.

### 3.4. Effect of Naringenin on the mRNA Expression Levels of CD86 and TLR2

**Figure 4:**
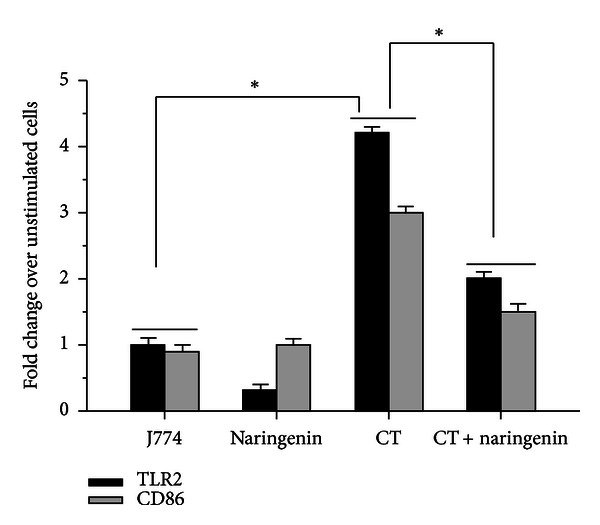
Naringenin reduces the transcriptional activation of TLR2 and CD86 by *C. trachomatis* in mouse J774 macrophages. Macrophages (3 × 10^6^ cells/mL) were left uninfected or infected with live *C. trachomatis* (3 × 10^5^ IFU/well). After 2-day infection of macrophages with *C. trachomatis* (CT), naringenin (1 *μ*g/mL) was added to macrophage cultures and 2 days later total RNA was extracted as described in Section 2. One step qRT-PCR was used to quantify the mRNA gene transcripts of TLR2 and CD86. *P < 0.05 is considered significant when compared to untreated cells (J774) and to cells treated with CT or CT + naringenin. Data shown is an average of triplicates run representative of two separate experiments.

As a further validation of our flow cytometric results, we next determine the effect of naringenin on the mRNA gene transcript expression levels of TLR2 and CD86 in *C. trachomatis* infected J774 macrophages. *C. trachomatis* enhanced the gene transcripts expression levels of TLR2 and CD86, which were both significantly (*P* < 0.05) downregulated (up to a 2-fold decrease) in the presence of naringenin (at 1 *μ*g/mL) (Figure 4). Combining these findings suggests that naringenin downregulates TLR2 and CD86 expression at both the protein and mRNA gene transcripts levels, thus underscoring its role in regulating *C. trachomatis* inflammation in macrophages.

### 3.5. *C. trachomatis* Uses the p38 MAPK Pathway to Induce Inflammatory Mediators

**Figure 5:**
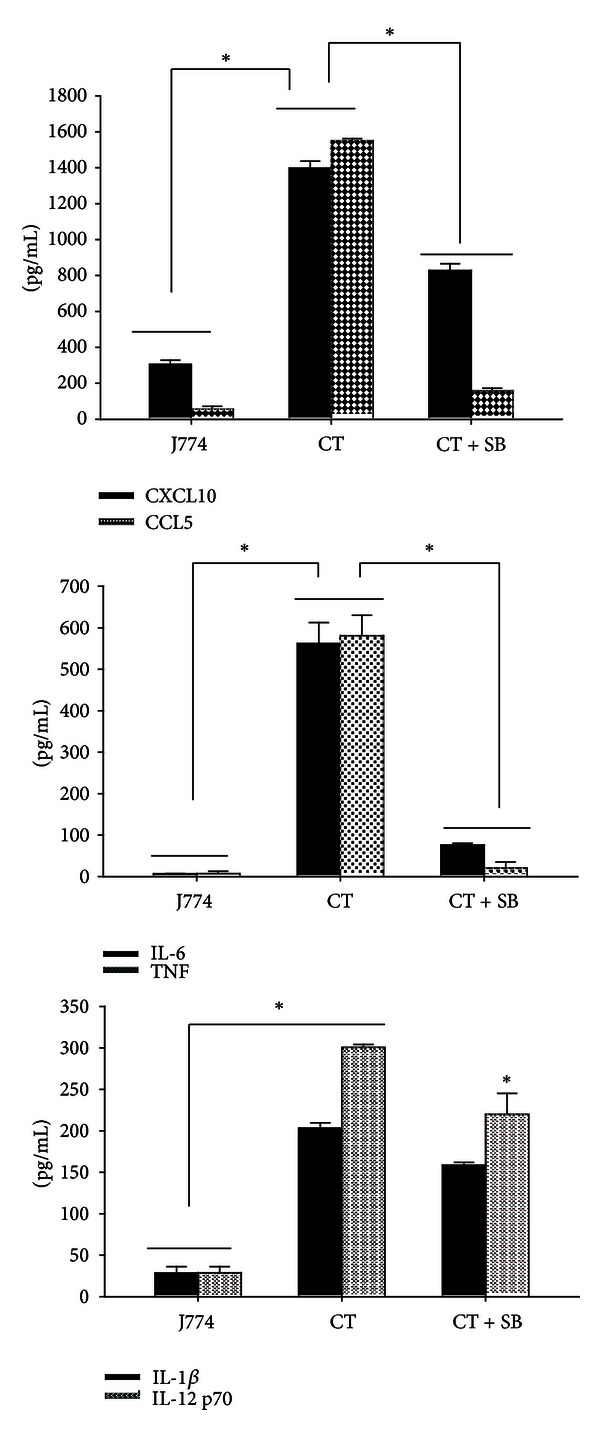
*C. trachomatis* employs the p38 MAPK pathway for production of inflammatory mediators in mouse J774 macrophages. Macrophages (10^6^ cells/mL) were seeded in 24-well plates in the presence and absence of the SB-203358 p38 inhibitor for 24 h, after which they were left uninfected or infected with live *C. trachomatis* (10^5^ IFU/mL). After 3 days following infection with *C. trachomatis* (CT), supernatants were collected and the levels of inflammatory mediators were determined by single ELISAs. *P < 0.05 is considered significant when compared to untreated cells (J774) and to cells treated with CT or CT + SB. Data shown is an average of duplicate run representative of two separate experiments.

Among the many MAPK pathways, strong link has been established between the p38 signaling pathway and inflammation [31]. Multiple studies have suggested that p38 is a key MAPK pathway that is activated by intracellular pathogen to induce inflammatory mediators [31–33]. To investigate if the p38 pathway is exploited by *C. trachomatis* for production of its concomitantly elicited inflammatory mediators, we treated J774 macrophages with a p38 specific inhibitor followed by quantification of randomly selected cytokines and chemokines in collected supernatants. With the exception of IL-1*β*, our result shows that the levels of IL-6, IL-12p70, TNF, CCL5, and CXCL10 were significantly reduced (*P* < 0.05) when macrophages were treated with the p38 inhibitor (Figure 5), suggesting that this pathway is used by *C. trachomatis* for their production by macrophages.

### 3.6. Naringenin Downregulates *C. trachomatis* Phosphorylation of p38 MAPK

**Figure 6:**
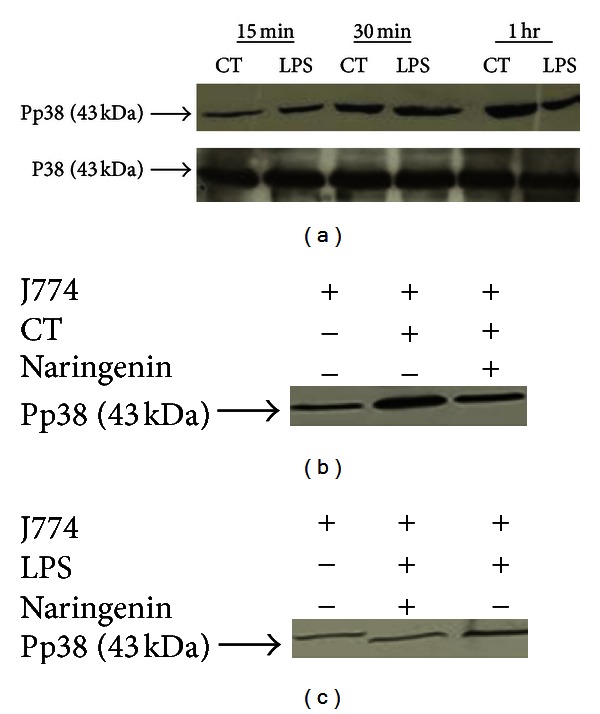
Naringenin downregulates the phosphorylation of p38 MAPK in *C. trachomatis* infected mouse J774 macrophages. Macrophages (3 × 10^6^ cells/well) were seeded in 6-well plates and then infected with *C. trachomatis* (3 × 10^5^ IFU/well) or LPS (3 *μ*g/well). After infection of macrophages, protein lysates were collected as indicated in Section 2. The presence of total p38 (p38) (internal control) and phosphorylated p38 (Pp38) was determined by western blotting. Shown are the band intensities for internal control and pp38 at different time points for macrophages treated with *C. trachomatis* (CT) and LPS (a). Blots shown in (b) and (c) were developed 1 h after exposing macrophages to CT or LPS in the presence and absence of naringenin. The 43 kDa Pp38 and p38 proteins were determined from a known biotinylated protein ladder.

Given that p38 MAPK mediates, in part, the production of inflammatory mediators by *C. trachomatis* infected macrophages, we investigated if this pathway may be used by naringenin to exert its anti-inflammatory effect in macrophages. Therefore, we first determined that indeed *C. trachomatis* could induce the phosphorylation of p38 MAPK in J774 macrophages for the production of its inflammatory mediators. Our time-kinetics experiment shows that *C. trachomatis* infected macrophages expressed the highest p38 phosphorylation at 60 min (Figure 6(a)). However, in the presence of naringenin, the phosphorylation of p38 reduced as indicated by the reduced band intensity (Figure 6(b)). Similarly, LPS induced the phosphorylation of p38 at 60 min of stimulation, but naringenin reduced its ability to induce phosphorylation of p38 (Figure 6(c)). Overall, our results show increased phosphorylation of p38 MAP kinase in *C. trachomatis* infected macrophages, which was downregulated by naringenin, suggesting a potential downstream mechanism for naringenin to regulate inflammatory mediators.

## 4. Discussion

Inflammatory responses to *C. trachomatis* are initiated and sustained by actively infected host cells including epithelial cells and resident macrophages [4]. The influx of inflammatory cells in pathogen-induced diseases can be either beneficial or detrimental to the host [28]. Therefore, immunointervention strategies that can reduce the influx of inflammatory cells in a beneficial fashion could potentially impact the pathogenesis of diseases. Along with other controlling strategies, our laboratory is also interested in evaluating anti-inflammatory molecules to control *C. trachomatis* inflammation. Previously we have shown that the anti-inflammatory cytokines, IL-10, downregulate essential inflammatory mediators produced by epithelial cells infected with live *C. trachomatis* [9]. In the present paper we explored the natural flavonoid, naringenin, as a potential anti-inflammatory agent to regulate inflammatory mediators produced by *C. trachomatis* infected macrophages. Among the numerous structural diversities, we selected naringenin based on its abundance in nature and potential application in medicine. The following observations were made here: (1) by multiplex ELISA a spectra of cytokines and chemokines, which may perpetuate an early *C. trachomatis* inflammation, were revealed, (2) naringenin downregulated cytokines and chemokines as produced by *C. trachomatis* infected macrophages, (3) naringenin downregulated TLR2 and TLR4 and also the CD86 costimulatory molecule on infected macrophages, and (4) naringenin inhibited the ability of *C. trachomatis* to phosphorylate p38 MAPK for production of its inflammatory mediators by macrophages.

Activation of immune cells, especially macrophages with microbial stimuli, influences the nature and progression of disease. In this study, analysis from *C. trachomatis* infected macrophages revealed increased levels of GM-CSF, IL-1*α*, IL-1*β*, IL-6, TNF, IL-12p70, and IL-10 after a 2-day infection, with TNF, IL-6, and IL-1*α* being more robustly produced (Figure 1(a)). Indeed this observation is of no surprise since cytokines are secreted at different magnitudes during the infection process. It is well reported that all secreted cytokines have their own specific role during the infection process [1, 4–8]. One plausible explanation for lower levels of IL-12p70, IL-10, IL-1*β*, and GM-CSF may be attributed to differences in the time kinetics for their optimum secretion during the infection process. Interestingly, this finding is in agreement with previous studies where lower levels of IL-10 were detected during *Borrelia* infection of human monocytes [34] and *C. trachomatis* infection of human epithelial cells and macrophages [9]. The heightened secretion of TNF, IL-6, and IL-1*α* by *C. trachomatis* infected macrophages may have some relevancy to the initiation of a *Chlamydia* inflammation. It has been demonstrated that IL-6, TNF, and IL-1*α* have crucial roles in increasing the intracellular adhesion molecule (ICAM) [4]. Infection of nonimmune host epithelial cells and resident tissue innate immune cells with *Chlamydia* results in an increase in adhesion molecules, whereby these molecules promote binding of small proteins such as chemokines on cell surfaces [4].

Chemokines are also produced during an infection to amplify the inflammation process. Chemokines play critical role attracting leukocytes to the site of infection, where the leukocytes presence can be seen either as beneficial or detrimental to the host. The main leukocytes that are recruited and attracted by chemokines during an early inflammatory process are macrophages and neutrophils [1–6]. Our result shows that *C. trachomatis* infected macrophages produced greater quantities of CCL4, CXCL10, CCL5, CXCL1, and CXCL5 (Figure 1(b)). The production levels of most chemokines are typically influenced by the type of cytokines present in the inflammatory milieu. The different profiles of chemokines produced by infected macrophages in this study correlated with the high levels of IL-6, TNF, and IL-1*α*. High levels of IL-6, TNF, and IL-1*α* apparently cause chemokines to stick to endothelial cell surfaces for efficient attraction mainly due to an increase in ICAM [4]. Overall, our results clearly demonstrate that the spectra of cytokines and chemokines produced by *C. trachomatis* infected macrophages may have significant roles in initiating its inflammatory process and thus pathogenesis of disease.

Naringenin has a broad-spectrum medicinal application against bacteria, parasitic, and viral infections. Lakshmi et al.'s [35] study showed an antifilarial activity of naringenin against the filarial parasite, *Brugia malayi* [35]. Naringenin was also shown to exhibit antimicrobial activity against pathogenic bacteria like *Listeria monocytogenes, Escherichia. coli O157:H7* and *Staphylococcus aureus* [36]. Similarly, an antiviral activity of naringenin was shown against herpes simplex virus type-1 (HSV), polivirus, parainfluenza virus type-3, and respiratory syncytial virus (RSV) [37]. Du and colleagues [21] demonstrated that naringenin regulates immune system function in a lung cancer infection model, where it reduced IL-4 but increased IL-2 and IFN-*γ* levels [21]. In a different study, Shi et al. [38] also showed that naringenin displayed an inhibitory role in allergen-induced airway inflammation by reducing IL-4, IL-13, CCL5, and CCL11 [38]. For the first time, in the present study we have shown that naringenin has an anti-inflammatory effect in an *in vitro C. trachomatis* infection model. Naringenin reduced in a dose-dependent manner the level of major inflammatory mediators secreted by *C. trachomatis* infected macrophages, which was not attributed to cell death. These studies suggest that naringenin has a broader immune-regulatory property in different disease models, especially inflammatory diseases.

In this study, we have clearly demonstrated that naringenin altered the levels of numerous cytokines and chemokines in *C. trachomatis* infected macrophages by its alteration of multiple inflammatory pathways. Induction of inflammatory pathway initially starts when invasive pathogens are recognized by cell surface receptor molecules such as TLRs in the host, followed by activation of various signaling pathways. It is well documented that *C. trachomatis* is recognized by TLRs specifically TLR2 and TLR4 on macrophages to induce secretion of inflammatory mediators, which can be either beneficial or detrimental to the host [29, 39]. Here in the present study we show enhanced expression of both TLR2 and TLR4 on *C. trachomatis* infected macrophages and whose expression levels were reduced by naringenin (Figures 3 and 4). Our study suggests the capacity of naringenin to inhibit the interaction of *C. trachomatis* with its upstream putative receptors to potentially mediate its anti-inflammatory effect in macrophages.

TLR-stimulated macrophages induce effectors of the adaptive immune system such as CD40, CD80, and CD86 to drive T-cell activation and proliferation. The CD28-mediated costimulatory signal can result in an enhanced T-cell proliferation and cytokine production which contributes to the development of various inflammatory diseases [40–42]. Our flow cytometry result demonstrates that *C. trachomatis* induced the expression of CD80 and CD86, however, with only CD86 expression being modulated by naringenin (Figures 3 and 4). Although we have not shown it in this study, but inhibiting CD80 and CD86 expression has a possibility to impair the activation of T cells and eventually blocking effectors of the adaptive immune system. Lim and coworkers documented significant reduction in the levels of IL-2 and IFN-*γ* when both CD80 and CD86 costimulatory molecules were inhibited confirming the key role played by costimulatory molecules in functional T-cell activation [43]. Weakened T-cell activation is directly associated with less interaction between antigen presenting (APC) cells and T cells. Thus, our data provides mechanical insights of *C. trachomatis* engulfment by macrophages as indicative by heightened expression of CD80 and CD86, which eventually contributes to the activation of adaptive immune responses.

Down-regulation of only CD86 expression in the presence of naringenin provides evidence for its broader capability in modulating inflammatory response during *C. trachomatis* infection. However, the perplexing question remains as to why naringenin inhibited CD86 but not CD80 expression even though both are costimulatory molecules highly needed for T-cell activation and also by which cell-to-cell binding forces depend on their recognition. It has been reported that treatment with CD80/86 blocking antibodies reduced the interaction force of cell : cell conjugates [43, 44]. Both CD80 and CD86 can bind to the T-cell stimulatory receptor CD28 [44] and to the inhibitory receptor CTLA4 [45]. CD86 appeared to strengthen APC : T-cell interactions more markedly than CD80 since higher force reduction was observed after blocking CD86 alone than that achieved by disrupting CD80 alone [44, 45]. Therefore, the ability of CD86 and not CD80 to induce stronger APC : T-cell interaction indicates its crucial ability in initiating immune responses.

Upon microbial recognition by TLRs, MAPK signaling pathways are activated to produce inflammatory mediators. Of the many MAPK pathways, p38 is considered to be an important pathway to induce inflammatory mediators during *C. trachomatis* infection [46]. Our inhibition study supports this idea, where in the presence of a p38 inhibitor the levels of IL-12p70, IL-6, TNF, CCL5, and CXCL10 (Figure 5) were significantly reduced suggesting that this pathway is employed by *C. trachomatis* to induce these respective inflammatory mediators. Furthermore, phosphorylation of p38 by *C. trachomatis* in macrophages in this study (Figure 6) underscores that it triggers this pathway for producing its concomitant inflammatory mediators. Of outmost significance, naringenin inhibited the ability of *C. trachomatis* to phosphorylate p38 in macrophages, suggesting possibly its attenuation of concomitantly produced cytokines and chemokines. Other investigators have reported that naringenin's inhibitory role in allergen airway infection is associated with its down-regulating the activation of the NF-*κ*B pathway via MAPK pathway [38]. In another study, it was also shown that naringenin manifested its anti-inflammatory functions *in vitro* by inhibiting NF*κ*B in macrophages [47, 48]. Shi et al. [38] also reported that naringenin can suppress mucous production by inhibiting NF*κ*B activity in a murine model of asthma [38]. Overall, our findings coupled with the above mentioned reports provide evidence that inflammatory signaling pathways including MAPK, especially p38 and NF*κ*B, are potential targets for naringenin anti-inflammatory effects.

*C. trachomatis* has a prolonged and unique developmental life cycle which takes 24–72 h for completion after entry into target cells. This process involves lysis and reinfection of cells by the released EBs [4] after binding to their cognate cell surface receptors. Reinfection reportedly is one of the major characteristics of *C. trachomatis* persistent infection [4, 31] contributing to the pathogenesis of disease. The ability of naringenin to reduce cell surface receptor expression and associated inflammatory signaling pathways 48 h after infection of cells with *C. trachomatis* is a testament of naringenin regulation of inflammatory mediators during the reinfection process. Even though we focused on selected cell surface receptors and signaling pathways in this study, we cannot dismiss the involvement of other receptors like the nucleotide binding site/leucine-rich repeat (NBS/LRR) protein, NOD2 that is recognized by *C. trachomatis* [49] (and our unpublished observation) or the NF*κ*B signaling pathway that reportedly mediates naringenin anti-inflammatory actions [38].

Admittedly, the precise mechanisms by which naringenin downregulates surface receptors and signaling pathways were not investigated here. Nevertheless, we cannot rule out the possibility that naringenin regulatory activity may be the direct consequences of its reducing the *C. trachomatis* infectious load in macrophages, ultimately resulting in less induction of inflammatory mediators. Indeed naringenin has been shown to have antibactericidal activity against several pathogenic bacteria [36]. Whether or not naringenin has anti-bactericidal activity against *C. trachomatis* in macrophages is the topic of our ongoing investigations.

In summary, most intracellular microorganisms including *C. trachomatis* prefer not to be targeted by regimens that impair their perpetuation in cells by inducing unwanted immune responses to amplify the disease progression. Therefore, in such scenarios, immunointervention approaches that focus on reducing any unwanted host immune response is attractive and can be viewed as alternative means to prevent or control severe inflammatory responses. Our findings presented here are the first, to our knowledge, to demonstrate that naringenin is an immunomodulator of inflammatory responses triggered by *C. trachomatis* in macrophages. Reduction of these inflammatory mediators by naringenin is mediated upstream by modulating TLR2, TLR4, and CD86 macrophage surface receptors and downstream via the p38 MAPK signaling pathway. More studies are warranted to further explore the *in vivo* relevancy of naringenin in controlling severe inflammatory responses that are induced not only by *C. trachomatis* but also by other similar pathogenic microorganisms.
